# Effect of Different Denture Base Materials and Changed Mouth Temperature on Dimensional Stability of Complete Dentures

**DOI:** 10.1155/2016/7085063

**Published:** 2016-04-07

**Authors:** Khalid A. O. Arafa

**Affiliations:** Dental Health Department, Faculty of Applied Medical Sciences, Al Baha University, P.O. Box 7273, Unit No. 2, Al Baha 65536-3047, Saudi Arabia

## Abstract

*Background*. Type of materials used in fabrication of denture base has an effect on dimension during denture base material processing and other factors related to clinical use.* Objective*. The study aims were to assess the dimensional stability including thermal changes of three different denture base materials.* Methods*. Ninety patients were selected to construct complete dentures with different denture base materials. They were randomly divided into three groups: group 1, patients with cobalt chrome metallic base; group 2, patients with heat curing acrylic resin fabricated by injection moulding technique; and group 3, patients with denture bases fabricated by conventional heat curing acrylic resin. The dimensional changes were assessed using digital caliper.* Results*. After the twelfth month, injection moulding acrylic resin had significantly the highest dimensional change followed by the conventional heat curing acrylic resin. There were no significant differences in the dimensions between the three types of denture base materials at normal mouth temperature, while, after hot tea drinking at 45°C, the dimensional change was significantly the highest in cobalt chrome metallic denture base group.* Conclusion*. Cobalt chrome metallic denture base has stable dimension compared to denture bases fabricated of acrylic resin but it was more affected by altered mouth temperature. The study was registered in the International Standard Randomized Controlled Trials Number (ISRCTN) registry with study ID (ISRCTN94238244).

## 1. Introduction

A good complete denture should provide esthetics, restore function, and be biocompatible with supporting and surrounding oral tissues. A successful denture should have dimensional stability in order to enhance chewing efficiency, increase patients' comfort, and prevent injury to the oral tissue [1]. During processing, dimensional changes of the acrylic denture base are affected by the type of material used in fabrication of denture base and other factors like polymerization shrinkage or stresses generated by cooling of flask [2]. Although acrylic resin is the most commonly used material in fabrication of denture base, it is dimensionally changed and distorted during acrylic processing and throughout clinical use [3]. These dimensional changes lead to inappropriate adaptation of the denture base to the oral tissue, reduced denture stability, and changes of the positions of the artificial teeth [4]. In addition to factors related to physical properties, processing procedures of denture base material, physiological and the anatomical conditions of patient's oral tissue also could affect the dimensional stability of denture base [5, 6]. Denture bases fabricated of cobalt chrome are characterized by dimensional stability regarding inherent properties but they could be affected by thermal changes in the oral environment [7]. Therefore, many researches aimed to compare dimensional stability of new denture base materials and processing techniques [8]. This study had two main aims; the first was to assess the dimensional stability of three different denture base materials over one year period of clinical use. The second aim was to assess the dimensional stability of three different denture base materials after consuming hot and cold beverages.

## 2. Methods

A quasi-clinical trial, parallel design, was conducted at the Faculty of Dentistry of Al-Azhar University in Egypt during 12 months (December 2014 to November 2015).

This study was approved by the Dental Health Department of the Faculty of Applied Medical Sciences, Al Baha University. The consents forms were filled by all participants. The right of the participants to withdraw at any time was explained and preserved during the study. The study was registered in the International Standard Randomized Controlled Trials Number (ISRCTN) registry with study ID (ISRCTN94238244).

The study was conducted on ninety patients. The patients were completely edentulous males at the age of 60 years or above. After exclusion of ineligible patients for whom the anterior-posterior dimensional measurements could not be standardized, the patients were distributed randomly using random tables. Thirty patients were included in each group, all treated with complete denture. The difference was in the denture base materials. The three groups received complete dentures fabricated by one of the following base materials (heat cure acrylic resin with conventional technique, heat cure acrylic resin with injection moulding technique, and cobalt chrome metallic).

The first group received complete denture fabricated by heat cure acrylic resin with conventional technique (Meliodent, Bayer Dental, Germany, batch number 54105L-2) curing cycles in acrylic furnace, at 165°F for 9 (acrylic furnace BEGO sommer, Germany, batch 226-433), the second group received complete denture fabricated by injection moulding technique of heat curing acrylic resin (Vertex ThermoSens, rigid, batch noxu373802, USA), and the third group received complete denture fabricated by cobalt chrome metallic denture base (BEGO metal denture base, Germany, batch 233-42), using the burnout furnace controlled electronically at (950–1200)°C fasting furnace (BEGO, Germany, batch 239270). The laboratory procedures were conducted according to manufacturer's instructions.

**Figure 1:**
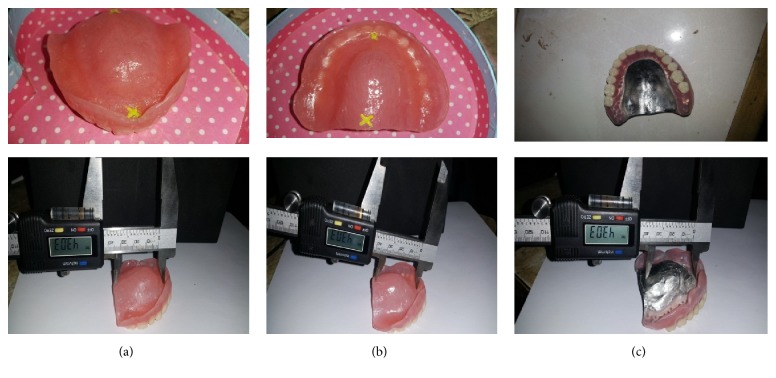
The picture demonstrated points A and B on the fitting surface of dentures.

Figure 1 shows the points used for distance measurement on dentures made of heat cure acrylic resin with conventional technique (a), heat cure acrylic resin with injection moulding technique (b), and cobalt chrome metallic material (c).

The commonly used method of assessing accuracy of denture dimension included measuring between set points on the denture base using caliper. During the last three decades, it was found that 60% of papers tackling dimensional stability of denture bases materials used microscopy and 25% utilized calipers instrument [9]. Furthermore, the use of calipers was proved to be a highly reliable instrument to assess oral vertical dimension [10].

For all the patients, the construction of complete dentures started by primary impression that was taken by alginate. Then, in the second visit secondary impression was taken by rubber base (Coltène Swiss quality for dentistry, batch 4160). In the third visit, the jaw relationship was recorded, and in the fourth visit a try in the waxed denture (Cavex, Holland, batch Z.A 990.01) was obtained, followed by construction of denture base in the lab. To assess the anterior-posterior dimensional changes, two different points are prepared in the fitting surface of the waxed denture (first point in the incisive papilla and second point in mid line of the postdam). Small crosses were marked with a carver on center of incisive papilla (point A), and the second point (B) was marked by small crosses in midline of the postdam (Figure 1). The length between A and B points was standardized in all dentures by using metallic bar. Therefore, in some cases the B point was located just anterior to the postdam, and when B point located outside the postdam, as indicated by metallic bar, the patient was excluded from this study. The distance between two points in the fitting surface was measured by dial caliper (brand name: Mitutoy, model number: Us_Ms00_13, model: 500 series, and place of origin: Japan) step measurement: a caliper with two jaws where one is fixed and the other is movable. Move the sliding jaw by pressing the thumb on the bump on the bottom. The caliper is used for reading of the distance between centers of the two points (unit of measurement is the millimeter with precision of 0.01). All groups with complete dentures were evaluated at baseline directly after insertion, and then they were evaluated after 4 months, 8 months, and lastly 12 months of clinical use. Measurement of dimensional change was calculated by subtracting distance between A and P points at baseline assessment from those at follow-up sessions. Measurement of dimensional changes due to altered mouth temperature was taken at normal patient mouth temperature, after drinking hot tea at 45°C and after taking cold drink at 5°C. This measurement was taken only once at separate follow-up session after 1 month of clinical use. Every patient was given one minute to drink cold beverage and 2 minutes to drink hot one. The temperature of the lab was set to 25°C and the measurement of dimensional change was taken directly after patients have finished drinking the beverage. The time span between hot and cold beverage was about one hour to allow for sufficient wash-out period. The thickness of 1.5–2 mm was adopted in all acrylic resin dentures and the thickness of 2 mm was used in all cobalt chromes dentures.

The data were collected using data collection sheet containing general patient information and measurements throughout a year of patients' follow-up. The data were then analyzed by computerized method (Statistical Package for Social Sciences) (SPSS version 20). The chi-square test was used to test differences in patients' categorical characteristics between the three groups. The paired *t*-test was used to detect differences between two acrylic resin groups and the control group of cobalt chrome regarding dimensional change in the same mouth temperature. Then ANOVA test followed by* ad hoc* Tukey's test was used to identify significant differences between the three groups for thermal changes. All values were tabulated as average of millimeter (mean) with standard deviation (SD). *p* values less than 0.05 were considered statistically significant.

## 3. Results

Ninety patients were recruited in this study; during the follow-up period 4 patients complained of pain and discomfort which had been released by little adjustment of the dentures. Only one patient was subjected to denture fracture in right molar area which has been repaired in the lab. The fracture line was so far from midline and assumed not to affect the measurement of dimensional accuracy. The study participants were homogeneous in the demographic characteristics such as age and gender distribution. The differences between study groups regarding demographic characteristics were not statistically significant (*p* > 0.05) (Table 1). There were no significant differences between anterior-posterior lengths in all groups at the base line assessment session, since they were constructed to be similar in all groups.

Regarding dimensional changes, the group with cobalt chrome denture base was considered as control group because metallic bases are known to be dimensionally stable in the constant mouth temperature. Dimensional expansions have occurred in both acrylic resin groups in comparison to cobalt chrome control group which remain dimensionally stable throughout one year of clinical use. Denture bases fabricated of heat curing acrylic resin by injection moulding technique were subjected to higher dimensional expansions than those fabricated by conventional technique. In comparison with cobalt chrome control group, the dimensional changes were always significantly higher in denture bases constructed by injection moulding technique after 4, 8, and 12 months of clinical use, while the dimensional changes which affected denture bases constructed by conventional technique show no statistically significant difference until the 12 months of clinical use (Table 2).

Concerning dimensional changes that followed altered mouth temperature, denture bases fabricated of heat curing acrylic resin by conventional technique show dimensional stability during thermal changes applied in follow-up sessions, while cobalt chrome denture bases showed significantly higher dimensional changes when compared to both types of heat curing acrylic resin. As a metallic material, it shrinks on cold temperature and expands with hot temperature. Denture bases made of acrylic resin by injection moulding technique showed insignificant slight shrinkage with cold drink (Table 3).

## 4. Discussion

The dimensional changes of the three denture base materials were varied, since heat curing acrylic resin fabricated by injection moulding technique was with low dimensional stability, while the cobalt chrome metallic denture base was dimensionally stable over one year of clinical service (4, 8, and 12 months). Furthermore, the study reported that the changes of mouth temperature have an effect on the denture base dimensions on cobalt chrome denture base. The cobalt chrome denture bases were more subjected to dimensional changes due to altered mouth temperature than acrylic resin denture bases. This study was a clinical trial aimed at assessing the dimensional stability of denture bases in intraoral environment over one year of clinical service, unlike the majority of previous studies that compared dimensional stability of denture base materials in laboratory setting. Effects of saliva and forces of mastication on dimensions of denture bases rather than the effect of polymerization shrinkage were mainly assessed over time in this study. M.-J. Kim and C.-W. Kim conducted a study to compare the effect of processing and immersion in artificial saliva on different denture base materials. They found that conventional resin group showed significantly largest dimensional changes after processing and immersion in artificial saliva for several weeks, while metallic base group showed significantly the smallest dimensional changes [11].

The dimensional expansion affecting acrylic resins during intraoral use could be attributed to known property of water sorption found in both types of acrylic resin [12, 13]. In laboratory setting, Young found the conventional technique more dimensionally accurate than injecting moulding technique using cobalt chrome as gold standard for comparisons. These findings were in agreement with the findings found by the current clinical study [14].

In another hand, a study conducted by Keenan et al. showed different findings, although it aimed to identify the dimensional changes related to heat curing techniques. The denture bases fabricated by injection moulding technique were found to have more expansion in vertical dimension than conventional heat curing technique. But this dimension was not assessed in the current study where only anterior-posterior dimensional changes were assessed [12]. Also a laboratory study conducted by Nogueira et al. found no significant horizontal dimensional changes between conventionally fabricated acrylic resin and those constructed by injecting moulding technique. However, they found significant dimensional changes at vertical dimensions caused mainly by polymerization shrinkage [15].

A clinical evaluation study conducted by Polychronakis et al. aimed to determine the dimensional changes on construction and in 5-year clinical service of acrylic resin complete dentures. They found initial shrinkage during insertion of dentures followed by compensation of this shrinkage during the first 3 months of clinical use. After that, expansion started to happen in the denture base of acrylic resin increasing with duration of clinical service. These findings were in agreement with the current study that found gradual expansion affecting acrylic resin base materials starting from 4-month follow-up period till one year of follow-up [16]. The anterior-posterior length is an important confounding factor in studies which aim to assess dimensional accuracy [11, 12]. Therefore, to adjust for confounding effect in this study, the length of distance between A and B points was made equal in all studied dentures. The thickness of denture base was also standardized to be 1.5–2 mm in acrylic resin dentures and 2 mm in cobalt chrome dentures.

The major limitation of this study is time of follow-up, since one year is considered as short follow-up period for complete denture wearers who are usually wearing dentures for several years [17]. The dimensional changes in mesiolingual and vertical directions were not assessed in this study which was able to generate more comprehensive understanding for dimensional stability of studied denture base materials.

## 5. Conclusion

Dimensional expansions have occurred in both types of acrylic resin denture bases especially denture bases fabricated by injection moulding technique, while cobalt chrome control group remains dimensionally stable. The change of mouth temperature was more tolerated by dentures produced from acrylic resin denture bases especially those fabricated by conventional technique.

## Figures and Tables

**Table 1:** Demographic characteristics of the patients that took part in the study.

Variable	Types of material			*p* value
	Acrylic resin fabricated by conventional technique Number (%)	Acrylic resin fabricated by injection moulding technique Number (%)	Chrome cobalt metallic Number (%)	
Gender				
Male	16 (53%)	15 (50%)	17 (57%)	0.31
Female	14 (47%)	15 (50%)	13 (43%)	
Age ** (**mean ± SD)	61 ± 3 years	60 ± 2 years	60 ± 2 years	0.43

**Table 2:** The dimensional changes (mean and standard deviation) of the three denture base materials over time.

Base material	Time					
	After 4 months		After 8 months		After 12 months	
	Mean	SD	Mean	SD	Mean	SD
Acrylic resin fabricated by conventional technique	1.52	0.014	1.56	0.018	1.59^*∗*^	0.02
Acrylic resin fabricated by injection moulding technique	1.58^*∗*^	0.019	1.6^*∗*^	0.022	1.63^*∗*^	0.027
Cobalt chrome metallic	1.5	0.01	1.5	0.01	1.5	0.01

^*∗*^Statistically significant.

**Table 3:** The dimensional changes of the three denture base materials by temperature changes (after one month of denture insertion).

Base material	Temperature					
	Normal mouth temperature		Hot tea at 45°C		Cold drink at 5°C	
	Mean	SD	Mean	SD	Mean	SD
Acrylic resin fabricated by conventional technique	1.5	0.01	1.5	0.01	1.5	0.01
Acrylic resin fabricated by injection moulding technique	1.5	0.01	1.5	0.01	1.48	0.009
Cobalt chrome metallic	1.5	0.01	1.6^*∗*^	0.019	1.43^*∗*^	0.004

^*∗*^Statistically significant.
